# Unlocking the potential: Expanding plastid transformation for beyond-organelle function

**DOI:** 10.1093/plcell/koad179

**Published:** 2023-06-23

**Authors:** Peng Liu

**Affiliations:** Assistant Features Editor, The Plant Cell, American Society of Plant Biologist, USA; Donald Danforth Plant Science Center, Saint Louis, MO 63146, USA

Plastids are a group of plant organelles that carry out specific biosynthetic and metabolic functions. All plastids differentiate from proplastids into one of several specialized organelles. These include chloroplasts (the site of photosynthesis), chromoplasts (which synthesize various pigments, e.g. in fruits and flowers), and amyloplasts (which store starch in root cells). Recent advances in plastid transformation technology have shown numerous advantages and provide an alternative approach to nuclear genome transformation. For instance, the presence of multiple copies of the plastid genome offers high transgene expression, the lack of heterochromatin in the plastid results in no transgene silencing, and the maternal inheritance of the plastid genome reduces the likelihood of transgene contamination by pollen (Bock 2015). In their study, **Sébastien Bélanger and colleagues (Bélanger et al. 2023)** demonstrate that plastid transformation can also be used to generate traits outside of the organelle's function.

To test the ability of a plastid-encoded DNA fragment to silence nuclear genes, the authors transformed transgenes corresponding to the nuclear *Phytoene Desaturase* (*PDS*) gene into the tobacco plastid genome. The first transgene expressed a partial *PDS* cDNA sequence from 2 convergent plastid promoters and consequently produced double-stranded RNA (dsRNA line). The other transgenes contained a partial *PDS* cDNA sequence in either sense or antisense orientation, driven by a plastid promoter to express sense RNA or antisense RNA (sense line and antisense line). The pigment gene *PDS* was chosen to monitor gene silencing as its dysfunction leads to a bleaching phenotype in normally green tissues. The antisense line did not exhibit any phenotype, whereas both the dsRNA and sense lines displayed bleaching phenotypes, indicating that *PDS* expression was repressed. The maternal inheritance of plastid transgenic traits was confirmed through reciprocal crosses.

The tobacco nuclear genome encodes 2 *PDS* genes, *PDS1* and *PDS2*, that share 99% sequence identity. Both *PDS1* and *PDS2* mRNAs showed significant reduction in the dsRNA line compared with the wild-type line, suggesting the bleached phenotype of the dsRNA line is a consequence of *PDS* mRNA knockdown. At a later developmental stage, pigment-deficient plants begin to lose their bleaching phenotype while the *PDS* mRNAs remain reduced. This indicates that the reversion of the bleaching phenotype is regulated by additional mechanisms independent of *PDS* transcript levels. Conversely, although the sense line had a similar pigment-deficient phenotype, no changes in *PDS* mRNA accumulation were observed, indicating a different mechanism such as translational-level regulation is involved in the sense line and has yet to be investigated.

High-resolution small RNA sequencing of seedlings with transformed plastids revealed abundant that matched the *PDS* sequence in the dsRNA line. Further analyses revealed that the majority of small RNAs on both RNA strands were 21-nt, and these small interfering RNAs (siRNAs) are generated in a phased manner, similar to the plant-specific class of phased secondary siRNAs (phasiRNAs). Because phasiRNAs are triggered by a microRNA and generated through iterative cleavage of dsRNA by Dicer-Like proteins (Liu et al. 2020), Dicer processing might be a major mechanism for protruding siRNAs from plastid-transgene RNAs. It remains to be understood what triggers the production of plastid-encoded *PDS* phasiRNAs or if these are possibly “triggerless.”

The next question raised was whether a nuclear-encoded complementary RNA is required for siRNA processing. To address this, the authors assessed small RNAs in 2 additional tobacco lines to express dsRNA against insect gene targets that are not present in the tobacco genome. One line expressed dsRNA using 2 convergent plastid promoters, while the other line expressed a hairpin dsRNA with a single promoter. In both cases, siRNAs were abundant and mapped to both RNA strands. Additionally, 21-nt phasiRNAs were the predominant siRNA species. The above results indicate the plastid-expressed dsRNA does not need the complementary nuclear-encoded RNAs to produce siRNAs.

This study shows that plastid-driven transgene expression can lead to the production of siRNAs that target nuclear-encoded mRNAs. The dsRNA likely enters the cytoplasm from the plastid and generates 21-nt phasiRNAs for gene silencing (Fig.). This finding will enable future high levels of siRNA production without self-silencing the source transgenes as a tool to modify plant or pathogen gene expression in potential applications of crop engineering.

**Figure.**
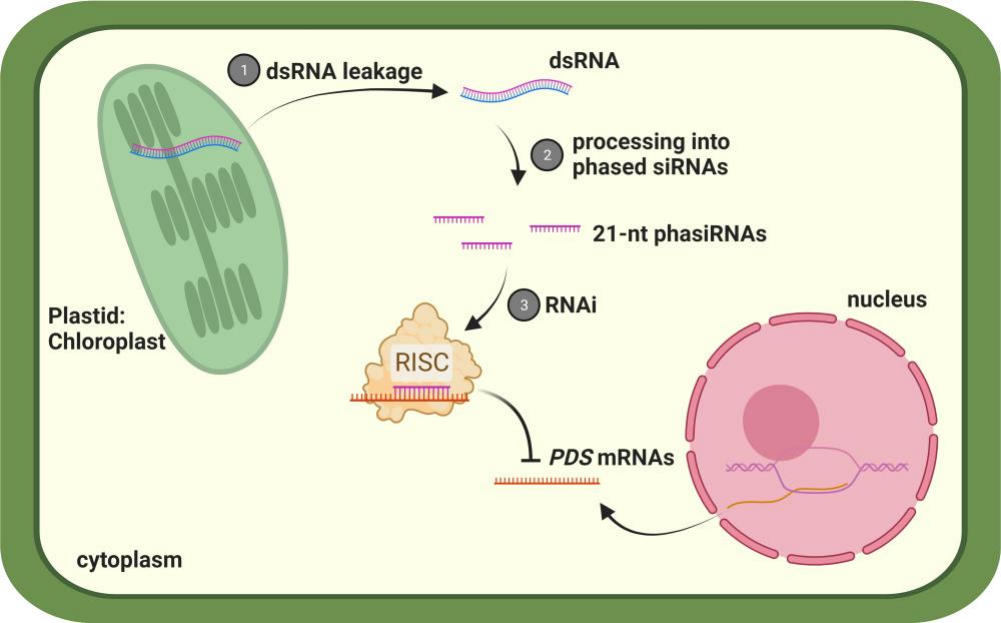
Plastid dsRNA triggers phasiRNA-mediated silencing of nuclear genes. The dsRNA is produced from the plastid transgene and leaks into the cytoplasm where it undergoes direct phasing resulting in the production of 21-nt phasiRNA. These phasiRNAs are then recruited into an RNA-induced silencing complex (RISC) to target and silence *PDS* mRNAs. Created by P. Liu with BioRender.com.
